# Rare disease models provide insight into inherited forms of neurodegeneration

**Published:** 2016

**Authors:** Philippa C. Fowler, Dwayne J. Byrne, Niamh C. O’Sullivan

**Affiliations:** UCD School of Biomolecular and Biomedical Sciences, UCD Conway Institute, University College Dublin, Dublin 4, Ireland

**Keywords:** Neurodegeneration, Hereditary Spastic Paraplegia, Endoplasmic Reticulum, Mitochondria, Animal Models of Rare Disease

## Abstract

Hereditary spastic paraplegias (HSPs) are a group of inherited neurodegenerative conditions characterised by retrograde degeneration of the longest motor neurons in the corticospinal tract, resulting in muscle weakness and spasticity of the lower limbs. To date more than 70 genetic loci have been associated with HSP, however the majority of cases are caused by mutations that encode proteins responsible for generating and maintaining tubular endoplasmic reticulum (ER) structure. These ER-shaping proteins are vital for the long-term survival of axons, however the mechanisms by which mutations in these proteins give rise to HSP remain poorly understood. To begin to address this we have characterized *in vivo* loss of function models of two very rare forms of HSP caused by loss of the ER-shaping proteins ARL6IP1 (SPG61) and RTN2 (SPG12). These models display progressive locomotor defects, disrupted organisation of the tubular ER and length-dependant defects in the axonal mitochondrial network. Here we compare our findings with those associated with more common forms HSP including: Spastin, Atlastin-1 and REEP 1 which together account for over half of all cases of autosomal dominant HSP. Furthermore, we discuss recent observations in other HSP models which are directly implicated in mitochondrial function and localization. Overall, we highlight the common features of our rare models of HSP and other models of disease which could indicate shared mechanisms underpinning neurodegeneration in these disorders.

## Text

Hereditary spastic paraplegias (HSPs) are a group of inherited neurodegenerative disorders characterised by the retrograde degeneration of the longest motor neurons (MNs) in the corticospinal tract, leading to muscle weakness and spasticity of the lower limbs.

Historically HSP was grouped into two classifications: ‘pure HSP’ exclusively exhibiting lower limb spasticity and ‘complicated HSP’ in which patients presented with additional symptoms including dementia, seizures and amyotrophy 1,2. Worldwide the prevalence of HSP ranges from <1 – 6 per 100,000, though some forms of the condition have been identified in only a few families^3^. HSPs are highly genetically diverse with traditional association studies and, more recently, whole exome sequencing identifying over 70 different spastic paraplegia gene (SPG) loci associated with HSP which can display autosomal dominant, autosomal recessive or in rare cases x-linked inheritances^4^,^5^. Despite this diversity, it is becoming clear that the most common cause of AD-HSP are mutations in genes encoding proteins that localise to and regulate the morphology and trafficking of intracellular membrane compartments, particularly the endoplasmic reticulum (ER)-shaping proteins.

## ER-shaping proteins and axonal health

ER-shaping proteins comprise intramembrane hairpin-loop domains by which they localise to and insert into the ER membrane. Spastin, Atlastin-1 and REEP1 are hairpin-loop domain containing ER-shaping proteins which account for ~50% of all autosomal dominant cases of HSP (AD-HSP). Spastin gene mutations cause spastic paraplegia 4 (SPG4) subtype, which represent about 40% of AD-HSP^6^. Spastin is an AAA-ATPase responsible for the regulation of microtubule dynamics in the outgrowth of developing axons and during their continued maintenance^7^–^10^. Mutations in the Atlastin-1 gene (SPG3A), the majority of which are missense^11^, account for approx. 10% of autosomal dominant cases of HSP. Atlastin-1 is a dynamin-related GTPase which mediates the homotypic fusion of ER tubule and the formation of three-way junctions^6^,^12^, key structures to the continuous dynamics of the peripheral ER network. Three-way junctions allow existing tubules to slide along existing opposing tubules to form new junctions in the ER while maintaining structural homeostasis^13^. The REEP1 gene encodes receptor expression-enhancing protein 1 whose function, beyond ER membrane shaping is poorly understood^14^. Mutations in this gene account for between 2-6% of AD-HSP and cause the SPG31 subtype of HSP^15^. More recently, mutations in two other ER-shaping protein families have been identified to cause rare forms of HSP: reticulon-2 (RTN2) has been linked to SPG12^16^ and ADP-ribosylation factor-like 6 interacting protein 1 (ARL6IP1) has been linked to SPG61^17^. Both reticulon-2 and ARL6IP1 function to generate and stabilise the highly curved membrane of the tubular ER^6^, ^18^. Cellular and *in vivo* studies consistently reveal that loss of ER-shaping proteins causes profound disruption to the organisation of the ER. Expression of an SPG3A mutant form of Atlastin-1 was found to alter ER-morphology in HeLa cells from a reticular to a more tubular organisation^17^ while loss of Atlastin from the *Drosophila* nervous system results in visible fragmentation of the ER^11^. REEP1 is typically caused by mutations which produce a truncated REEP1, one such mutation resulted in the translation of a truncated REEP which was unable to associate with microtubules and induced disrupted ER morphology^12^. In addition, expression of a known SPG4 mutant form of the M1 spastin isoform, p.K388R, was found to induce thickened, closely packed microtubules which associated with, and altered the distribution of, ER tubules^18^. Taken together, there is evidence that ER-shaping proteins, and the tubular ER that they generate, might have a crucial role in the maintenance of very long MNs. However, the mechanisms by which loss of these proteins give rise to axonal degeneration in HSP remain under-researched.

## *In vivo* models of rare forms of HSP

To begin to understand the role of ER-shaping proteins in MNs *in vivo* we have recently reported on models of HSP which we have generated and characterised in the fruit fly, *Drosophila melanogaster*^19^. Specifically, we have modelled SPG12 and SPG61 by knocking down expression of the *Drosophila* orthologs of RTN2 and ARL6IP1 (Rtnl1 and Arl6IP1 respectively). Gene knockdown models were selected as they likely reflect the loss-of-function genetic variants expressed in SPG12 and SPG61 patients. Mutations in RTN2 include a complete gene deletion and a frameshift mutation predicted to produce a highly truncated protein, suggesting that SPG12 acts via a haploinsufficiency mechanism^14^. The HSP-causing ARL6IP1 mutation detected in SPG61 is a 4 base pair deletion leading to a frameshift mutation^15^; therefore it is likely that SPG61 may act in a similar mechanism. Behavioural analysis revealed that these loss of ER-shaping protein flies display an age dependent and progressive loss of locomotor activity compared to controls, without a marked reduction in lifespan^19^. This degenerative phenotype is accompanied by fragmentation of the tubular ER at the distal ends of long motor neurons. Our models therefore recapitulate key features of HSP in human patients and provide the opportunity to investigate the role of ER-shaping proteins in MN function.

**Figure 1:**
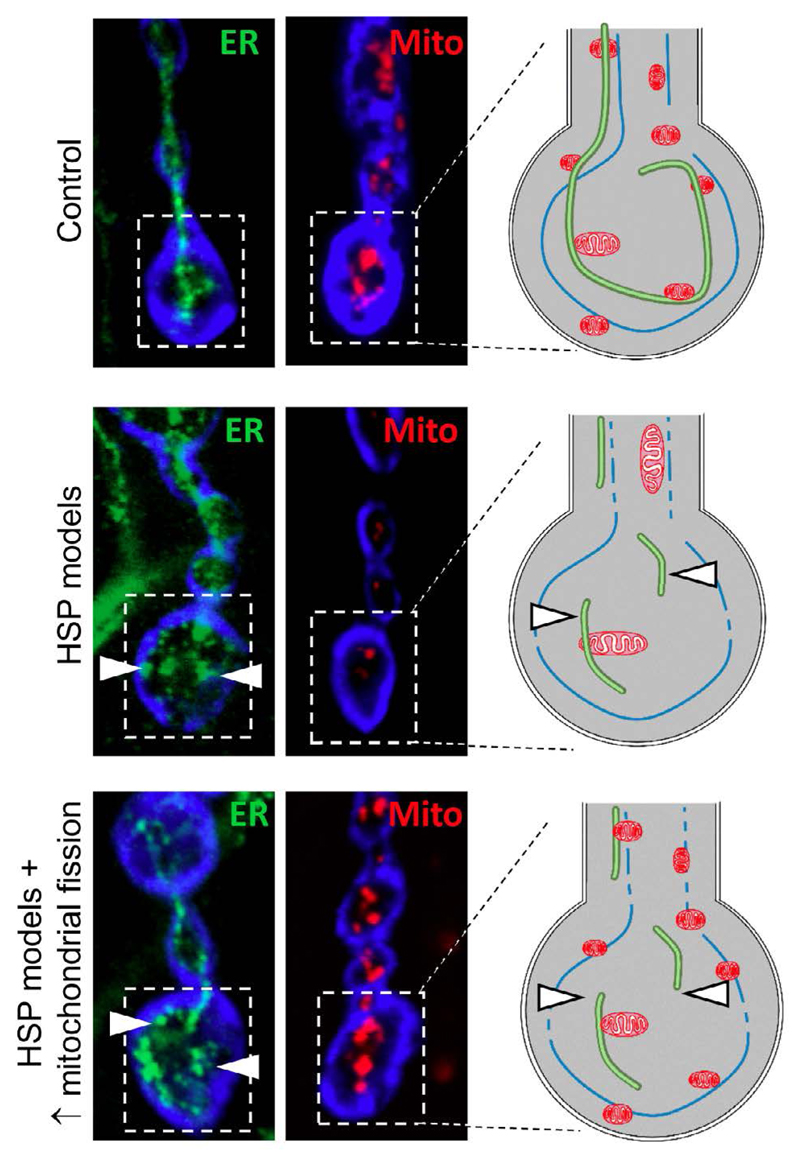
ER and mitochondrial disruption in the longest motor neurons of our *Drosophila* models of HSP. Confocal microscopic images showing tubular ER (Rtnl1::YFP; green) and mitochondria (mito::GFP; red) at the distal ends of long motor neurons. Schematic diagrams illustrate the ER fragmentation (arrowheads) and loss of mitochondria that we have identified in the long motor neurons in our model of HSP. While mitochondrial load is restored upon overexpression of the mitochondrial fission protein Drp1, the tubular ER remains fragmented.

The tubular ER is known to undertake many roles in neuronal axons including calcium homeostasis, lipid biogenesis and the regulation of mitochondrial fission, any or all of which may be impaired by loss of ER-shaping proteins. In our study, we have found that loss of ER-shaping proteins disrupts axonal mitochondrial organisation selectively in the longest *Drosophila* MNs. Specifically, mitochondria appear increasingly elongated within axon bundles and mitochondrial load within neuromuscular junction synaptic boutons is significantly reduced in loss of Arl6IP1 and loss of Rtnl1 *Drosophila* (Figure 1)^19^. Interestingly, these effects are only detected at the ends of the longest MNs while mitochondrial morphology and load in shorter MNs are unchanged from controls, pointing to a length-dependent sensitivity of MNs to loss of ER-shaping proteins. This phenotype is strikingly consistent with the degeneration of the longest upper MNs that is characteristic of HSP patients. It is currently not clear why disruption in our models occurs specifically in the distal ends of the longest MNs. Loss of the ER and mitochondria may contribute to neuronal dysfunction by disrupted calcium homeostasis, defective lipid biosynthesis or metabolomic disturbances. Long MNs, in which axonal cargo and signalling need to be maintained at great distances from the cell body, may be more susceptible to disruption in one or several of these cellular processes. Future investigation of functional defects in models of HSP should help to address this important question.

Together, the ER and mitochondria regulate a number of essential biological functions through highly specialised physical contacts known as mitochondria-associated endoplasmic reticulum membranes (MAMs). Importantly, these MAMs mediate mitochondrial fission in which the ER functions to mark mitochondrial constriction sites prior to the recruitment of mitochondrial fission factors such as dynamin related protein 1 (Drp1)^20^. We observed a striking disruption to the ER network in response to loss of Arl6IP1, accompanied by mitochondrial network organization defects; therefore we proposed that these changes could occur through altered ER-mitochondrial interactions and subsequent defects in mitochondrial fission dynamics. To test this hypothesis, we investigated whether increasing mitochondrial fission by genetically increasing expression Drp1 could restore mitochondrial organisation and modulate neurodegeneration in Rtnl1 and Arl6IP1 *Drosophila* models of HSP. We found that overexpression of Drp1 restored axonal mitochondrial morphology and mitochondrial load within terminal synaptic boutons of both Rtnl1 and Arl6IP1 *Drosophila* models of HSP (Figure 1). In contrast, overexpression of Drp1 does not restore ER fragmentation in these models. Importantly, the behavioural phenotypes caused by loss of ER-shaping proteins were ameliorated by Drp1 overexpression. Taken together, these results propose a role for ER-shaping proteins in mitochondrial network organization *in vivo* and suggest that impaired mitochondrial organization may be a common mechanism underpinning these rare forms of HSP. Furthermore, increasing mitochondrial fission may offer a novel avenue for therapeutic intervention in two distinct models of HSP.

## Similarities between rare and common forms of HSP

While our Rtnl1 and Arl6IP1 models of HSP represent very rare forms of disease, (identified in just 4 and 1 families respectively^14^,^15^), given that they are members of the larger ER-shaping group of proteins they likely also inform about the more common forms of HSP due to loss of Spastin, Atlastin-1 or REEP1. Several lines of evidence suggest that disrupted mitochondrial morphology, distribution and trafficking are common features of loss of Spastin, Atlastin-1 or REEP1. A mouse model of SPG4, caused by a loss of function mutation in spastin, resulted in disrupted anterograde (i.e. towards the distal ends of neurons) axonal transport of mitochondria^21^. Separately, olfactory mucosal cells obtained from a patient with Spastin mutations and differentiated to an adherent cell culture were found to exhibit a reduction in the presence of mitochondria at the cell periphery^22^. Similarly, the fast axonal transport of mitochondria was also found to be disrupted in forebrain neurons differentiated from iPSCs generated from SPG3A patient fibroblasts with mutations in Atlastin-1^23^ and highly elongated mitochondria were reported in skin fibroblasts from a SPG31 (REEP1) patient^13^. Finally, mitochondria from several patient samples have been found to be bioenergetically impaired^13^,^24^. These studies implicate defective mitochondrial transport, organisation or distribution as a common feature of HSP associated with loss of ER-shaping proteins. In addition to mitochondrial abnormalities that arise from mutations in ER-shaping proteins, there are also additional genes which are directly involved in mitochondrial function. This includes SPG7 which is caused by mutations in the Paraplegin gene and encodes a m-AAA protease responsible for mitochondrial protein quality control^25^. As well as this, SPG13 arises due to mutations in the gene encoding the mitochondrial chaperone HSP60^26^.

Mutations in proteins directly involved in mitochondrial transport are also known to cause HSP. KIF5A is a member of the kinesin heavy chain (KHC) family which act as microtubule motor proteins involved in the transport of membranous organelles. Mutations in KIF5A cause Charcot-Marie Tooth Disease Type 2 (CMT2) and SPG10^27^,^28^. Recently, KIF5A was shown to be required for axonal maintenance and mutant KIF5A zebrafish exhibit axonal degeneration and complex spastic behaviours reminiscent of human patients with KIF5A mutants. KIF5A mutants were shown to exhibit a significant reduction in axonal mitochondrial number while synaptic vesicle and lysosomal load were indistinguishable from wildtype. Mitochondrial motility in mutant axons was also significantly impaired. Mitochondrial deficiencies in this model were shown to precede behavioural abnormalities and axonal degeneration suggesting that impaired mitochondrial density and mobility contribute to, rather than result from, axonal degeneration^29^. The observation of altered mitochondrial localisation across multiple SPG models may provide a mechanism common to multiple forms of HSP. Furthermore, this offers support for our hypothesis that restoration of mitochondrial distribution may offer therapeutic potential.

Together these data suggest that disruption to the neuronal mitochondrial network, either directly via mitochondrial mutations or indirectly via disrupted tubular ER, underpins much of the axonal degeneration associated with HSP. Altered mitochondrial distribution could disrupt the bioenergetics of different cellular compartments, given the peculiar morphology of neurons with long axons and the requirement for energy at axonal terminals this may cause failure of normal neuronal function. This work illustrates how models of very rare forms of disease may provide novel insights into the molecular mechanisms underpinning HSP more generally and suggest novel targets for therapeutic investigation for these currently untreatable neurodegenerative disorders.

## References

[1] Harding AE (1983). Classification of the hereditary ataxias and paraplegias. Lancet.

[2] Helbig KL, Hedrich UB, Shinde DN, Krey I, Teichmann AC, Hentschel J (2016). A recurrent mutation in KCNA2 as a novel cause of hereditary spastic paraplegia and ataxia. Annals of neurology.

[3] Ruano L, Melo C, Silva MC, Coutinho P (2014). The global epidemiology of hereditary ataxia and spastic paraplegia: a systematic review of prevalence studies. Neuroepidemiology.

[4] Lo Giudice T, Lombardi F, Santorelli FM, Kawarai T, Orlacchio A (2014). Hereditary spastic paraplegia: clinical-genetic characteristics and evolving molecular mechanisms. Experimental neurology.

[5] Hu J, Shibata Y, Zhu PP, Voss C, Rismanchi N, Prinz WA (2009). A class of dynamin-like GTPases involved in the generation of the tubular ER network. Cell.

[6] Roll-Mecak A, Vale RD (2005). The *Drosophila* homologue of the hereditary spastic paraplegia protein, spastin, severs and disassembles microtubules. Current biology CB.

[7] Roll-Mecak A, Vale RD (2008). Structural basis of microtubule severing by the hereditary spastic paraplegia protein spastin. Nature.

[8] Errico A, Ballabio A, Rugarli EI (2002). Spastin, the protein mutated in autosomal dominant hereditary spastic paraplegia, is involved in microtubule dynamics. Human molecular genetics.

[9] Wood JD, Landers JA, Bingley M, McDermott CJ, Thomas-McArthur V, Gleadall LJ (2006). The microtubule-severing protein Spastin is essential for axon outgrowth in the zebrafish embryo. Human molecular genetics.

[10] Sanderson CM, Connell JW, Edwards TL, Bright NA, Duley S, Thompson A (2006). Spastin and atlastin, two proteins mutated in autosomal-dominant hereditary spastic paraplegia, are binding partners. Human molecular genetics.

[11] Orso G, Pendin D, Liu S, Tosetto J, Moss TJ, Faust JE (2009). Homotypic fusion of ER membranes requires the dynamin-like GTPase atlastin. Nature.

[12] Park SH, Zhu PP, Parker RL, Blackstone C (2010). Hereditary spastic paraplegia proteins REEP1, spastin, and atlastin-1 coordinate microtubule interactions with the tubular ER network. The Journal of clinical investigation.

[13] Goizet C, Depienne C, Benard G, Boukhris A, Mundwiller E, Sole G (2011). REEP1 mutations in SPG31: frequency, mutational spectrum, and potential association with mitochondrial morpho-functional dysfunction. Human mutation.

[14] Montenegro G, Rebelo AP, Connell J, Allison R, Babalini C, D’Aloia M (2012). Mutations in the ER-shaping protein reticulon 2 cause the axon-degenerative disorder hereditary spastic paraplegia type 12. The Journal of clinical investigation.

[15] Novarino G, Fenstermaker AG, Zaki MS, Hofree M, Silhavy JL, Heiberg AD (2014). Exome sequencing links corticospinal motor neuron disease to common neurodegenerative disorders. Science.

[16] Yamamoto Y, Yoshida A, Miyazaki N, Iwasaki K, Sakisaka T (2014). Arl6IP1 has the ability to shape the mammalian ER membrane in a reticulon-like fashion. The Biochemical journal.

[17] Rismanchi N, Soderblom C, Stadler J, Zhu PP, Blackstone C (2008). Atlastin GTPases are required for Golgi apparatus and ER morphogenesis. Human molecular genetics.

[18] Connell JW, Lindon C, Luzio JP, Reid E (2009). Spastin couples microtubule severing to membrane traffic in completion of cytokinesis and secretion. Traffic.

[19] Fowler PC, O’Sullivan NC (2016). ER-shaping proteins are required for ER and mitochondrial network organization in motor neurons. Human molecular genetics.

[20] Friedman JR, Lackner LL, West M, DiBenedetto JR, Nunnari J, Voeltz GK (2011). ER tubules mark sites of mitochondrial division. Science.

[21] Kasher PR, De Vos KJ, Wharton SB, Manser C, Bennett EJ, Bingley M (2009). Direct evidence for axonal transport defects in a novel mouse model of mutant spastin-induced hereditary spastic paraplegia (HSP) and human HSP patients. Journal of neurochemistry.

[22] Abrahamsen G, Fan Y, Matigian N, Wali G, Bellette B, Sutharsan R (2013). A patient-derived stem cell model of hereditary spastic paraplegia with SPAST mutations. Disease models & mechanisms.

[23] Zhu PP, Denton KR, Pierson TM, Li XJ, Blackstone C (2014). Pharmacologic rescue of axon growth defects in a human iPSC model of hereditary spastic paraplegia SPG3A. Human molecular genetics.

[24] McDermott CJ, Taylor RW, Hayes C, Johnson M, Bushby KM, Turnbull DM (2003). Investigation of mitochondrial function in hereditary spastic paraparesis. Neuroreport.

[25] Casari G, De Fusco M, Ciarmatori S, Zeviani M, Mora M, Fernandez P, De Michele G, Filla A, Cocozza S, Marconi R (1998). Spastic paraplegia and OXPHOS impairment caused by mutations in paraplegin, a nuclear-encoded mitochondrial metalloprotease. Cell.

[26] Hansen JE, Casaburi R (2002). Mitochondrial disorders and exertional intolerance: controversy continues. American journal of respiratory and critical care medicine.

[27] Reid E, Kloos M, Ashley-Koch A, Hughes L, Bevan S, Svenson IK (2002). A kinesin heavy chain (KIF5A) mutation in hereditary spastic paraplegia (SPG10). American journal of human genetics.

[28] Liu YT, Laura M, Hersheson J, Horga A, Jaunmuktane Z, Brandner S (2014). Extended phenotypic spectrum of KIF5A mutations: From spastic paraplegia to axonal neuropathy. Neurology.

[29] Campbell PD, Shen K, Sapio MR, Glenn TD, Talbot WS, Marlow FL (2014). Unique function of Kinesin Kif5A in localization of mitochondria in axons. The Journal of neuroscience: the official journal of the Society for Neuroscience.

